# The Role of Polymerase Chain Reaction (PCR) and Quantification Cycle Values in the Diagnosis of *Pneumocystis jirovecii* Pneumonia

**DOI:** 10.3390/jof11080557

**Published:** 2025-07-28

**Authors:** Tal Abramovich, Maya Korem, Rottem Kuint, Ayelet Michael-Gayego, Jacob Moran-Gilad, Karen Olshtain-Pops

**Affiliations:** 1 Faculty of Medicine, Hebrew University of Jerusalem, Jerusalem 91120, Israel; 2 Department of Clinical Microbiology and Infectious Diseases, Hadassah Medical Center, Jerusalem 91120, Israel; 3 Institute of Pulmonary Medicine, Hadassah Medical Center, Jerusalem 91120, Israel; 4 Clinical Microbiology Laboratory, Department of Clinical Microbiology and Infectious Diseases, Hadassah Medical Center, Jerusalem 91120, Israel

**Keywords:** *pneumocystis jirovecii*, pneumonia, (PCR) polymerase chain reaction

## Abstract

**Introduction**: This study aimed to assess the accuracy of real-time polymerase chain reaction (PCR) as a diagnostic tool for *Pneumocystis jirovecii* pneumonia (PCP) in immunocompromised patients and evaluate the applicability of quantification cycle (Cq) data for PCP diagnosis. **Methods**: Clinical and laboratory data were collected from medical records of 96 immunocompromised patients hospitalized at the Hadassah hospital from 2018 to 2022, for lower respiratory tract infection. PCP diagnosis was independently categorized by two infectious disease specialists, blinded to PCR results, as either “definite” (confirmed by microscopic identification of *P. jirovecii*) or “probable” (compatible clinical data and negative microscopy). Clinical characteristics, PCR test performance, and Cq values were then compared between these PCP diagnostic groups and a control group of 85 patients who underwent bronchoscopy for indications unrelated to *P. jirovecii* infection. **Results**: The PCR test was found to be highly reliable for diagnosing PCP, with high sensitivity and specificity (93.1%, 98.7%, respectively), a positive predictive value (PPV) of 96.4%, a negative predictive value (NPV) of 97.1%, a negative likelihood ratio of 0.71, and a positive likelihood ratio of 46.5. A Cq cutoff value of 21.89 was found to discriminate between probable PCP and definite PCP. In addition, patients with probable PCP had lower in-hospital mortality than those with definite PCP or no PCP. **Conclusions**: PCR offers a promising approach for diagnosing PCP in immunocompromised patients with negative respiratory microscopy results. While further research may be warranted, its use may allow for more timely treatment and potentially improved outcomes.

## 1. Introduction

*Pneumocystis jirovecii* (previously known as *carinii*) pneumonia (PCP), a life-threatening infection caused by the fungus *P. jirovecii* (PJ), primarily affects immunocompromised individuals, such as those with HIV/AIDS or organ transplants [1,2]. Diagnosis is challenging due to non-specific clinical and radiographic findings, and relies on microscopic examination of lung tissue or bronchoalveolar lavage (BAL) fluid. Fluorescein-conjugated monoclonal antibody or Gomori-methenamine silver staining are gold-standard methods, but can yield false negative results, especially in non-HIV immunosuppressed patients with low fungal burdens [1,2].

Polymerase chain reaction (PCR) is a highly sensitive and specific technique for detecting *P. jirovecii* DNA in respiratory samples, offering an advantage in diagnosis [3,4]. However, there is limited standardization among laboratories regarding the method and gene target of the PCR test. In addition, a positive PCR result alone cannot differentiate between active *P. jirovecii* infection and colonization. Colonization refers to the presence of an organism or its DNA in human tissue without causing signs or symptoms of infection. It is important to differentiate between these two conditions in order to avoid unnecessary antibiotic treatment in cases of mere colonization. Due to this drawback, The European Organization for Research and Treatment of Cancer and the Mycoses Study Group Education and Research Consortium (EORTC/MSG) [5] added PCR detection of *P. jirovecii* DNA in respiratory specimens as a criteria for “probable” PCP but not “proven” PCP. More data regarding the sensitivity and specificity of PJ PCR is needed.

Several studies suggest that PCR cycle of quantification (Cq) values (colloquially termed C*_T_* values), which are inversely proportional to the amount of *P. jirovecii* DNA present, may help distinguish between infection and colonization [4,6,7]. Despite this potential, the precise clinical utility of PCR, and particularly the use of Cq values in diagnosing PCP, requires further investigation.

This study aims to evaluate the diagnostic accuracy of real-time quantitative PCR (qPCR) for PCP in the context of routine clinical practice, and to assess the utility of Cq values in distinguishing between diagnostic groups.

## 2. Methods

### 2.1. Study Design

We conducted a retrospective study that included an analysis of clinical and laboratory data, including PCP diagnostic tests, from the electronic medical records of patients suspected of having PCP compared to controls.

The study group consisted of 96 immunocompromised patients who were hospitalized at the Hadassah Hospital between 2018 and 2022 with a diagnosis of lower respiratory tract infection, underwent diagnostic bronchoscopy (*n* = 94) or sputum induction (*n* = 2), and had respiratory samples tested for PJ using PCR and cytological staining (Grocott–Gomori methenamine silver).

To analyze the performance of PCR, we used a control group that included 85 ambulatory patients who underwent bronchoscopy for non-infectious reasons, provided in detail in Section 3, and whose BAL fluid was available for PJ testing by PCR.

### 2.2. Laboratory Methods

*Pneumocystis jirovecii* (PJ) staining was performed on bronchoalveolar lavage (BAL) specimens from patients with suspected PCP, following our hospital’s standard protocol. Centrifuged specimens were placed on slides, stained using Grocott–Gomori methenamine silver [5] and examined by experienced pathologists specializing in BAL analysis.

PCR testing—Following the extraction of DNA from respiratory samples (sputum or BAL fluid) using the easyMAG automated system (bioMerieux, Marcy l’Etoile, France), the presence of PJ DNA was assessed using a Taqman real-time PCR assay, based on previously described primers [8]. In short, the target gene for the reaction was the mitochondrial large subunit (mtLSU) ribosomal RNA gene of PJ. The forward primer (mtLSU FWD: 5′–TGG TAA GTA GTG AAA TAC AAA TCG G–3′ [start = 178, stop = 203]) and the reverse primer (mtLSU REV: 5′–ACT CCC TCG AGA TAT TCA GTG C–3′ [start = 277, stop = 299]) served to identify the gene. During each run, a PJ DNA positive control and a template negative control were included. Cq values were recorded for all positive samples, with a positive cut off Cq value ≤ 37.

The qPCR assay was calibrated using a synthetic DNA fragment used as the positive control. This sequence was synthesized by a professional laboratory according to the reference mentioned above [8] (Sequence: CTC ACT TGT AGA ACG GTG ATC AGC CTG TGC TCT AGA GCC TGA TAG TTG AGC GAT ACA CAC TGG TAA GTA GTG AAA TAC AAA TCG GAC TAG GAT ATA GCT GGT TTT CTG CGA AAA TTG TTT TGG CAA ATT GTT TAT TCC TCT AAA AAA TAG TAG GTA TAG CAC TGA ATA TCT CGA GGG AGT TGA TCG TTG AAG TCG ACC TAC ATC GAG TGC GCA CTA TCA AGA GTG TTC CAG TCA CGC GAT). A calibration curve was constructed following testing of known fragment copy numbers ranging from 1 × 10^0^ to 1 × 10^9^. The calibration curve was run in parallel to the testing of clinical samples and Cq values were converted to gene-target copy numbers accordingly [9].

### 2.3. Clinical and Laboratory Data

Demographic, clinical, and laboratory data were collected from patients’ medical records (Table 1). Laboratory data included PJ staining results from BAL samples, PJ PCR results, and Cq values.

### 2.4. Definitions

PCP was defined by two independent infectious diseases specialists as “definite,” “probable,” or “No PCP”; according to the following criteria:

Definite—suggestive clinical and/or radiographic findings +positive PCP microscopy.

Probable—suggestive clinical and radiographic findings with negative PCP microscopy + response to anti-PCP therapy, and no alternative diagnosis (without reference to the PCR result).

“No PCP”—not fitting the criteria for probable or definite PCP.

These criteria are based on international guidelines which are in agreement that ‘definite PCP’ is defined when the organism is identified by specific staining methods [10,11]. The definition of ‘probable PCP’ was based on the European AIDS Society guidelines [11].

The diagnostic accuracy of the PCR results was then defined based on the following metrics: sensitivity, specificity, PPV, NPV, positive likelihood ratio and negative likelihood ratio. In addition, we attempted to identify a cutoff Cq value that could distinguish between the diagnostic groups mentioned above.

### 2.5. Statistical Analysis

Pearson’s chi-squared test and Fisher’s exact test were used to test the association between two categorical variables.

To test the association between continuous variables, we used the Kruskal–Wallis test, *t*-test, and Mann–Whitney test.

To determine an optimal Cq value to distinguish between the different groups of diagnoses, we used a ROC (receiver operating characteristic) curve analysis.

All tests were two-sided, and *p*-values lower than 0.05 were considered statistically significant.

## 3. Results

The clinical characteristics of both the study group and the control group, including underlying illnesses, immunosuppressive therapy, disease severity, clinical presentation, outcomes and preventive treatment for PCP, are shown in Table 1.

The control group consisted of 85 patients who underwent bronchoscopy as outpatients, none of whom were suspected clinically of having PCP. Underlying illnesses are summarized in Table 1. As shown in the table, the frequencies of solid and hematologic malignancies, autoimmune diseases, chronic lung disease and dialysis were comparable between the groups. In contrast, the frequency of HIV, solid organ transplant, and treatment with steroids and chemotherapy was higher in the study group. Seven (8%) patients in the control group did not have underlying diseases. The indications for performing bronchoscopy in the control group were as follows: 38 (45%) had pulmonary nodules or a lung mass, 12 (14%) had interstitial lung disease, 10 (12%) had infected bronchiectasis, 9 (10%) had hemoptysis, and 8 (9%) had chronic cough.

The study group was further divided into subgroups according to the results of the different diagnostic tests, as detailed in Section 2. A total of 9 were defined as “definite PCP” due to a positive PJ stain on microscopic examination, 20 were defined as “probable”, and 67 as “No PCP”.

**Figure 1:**
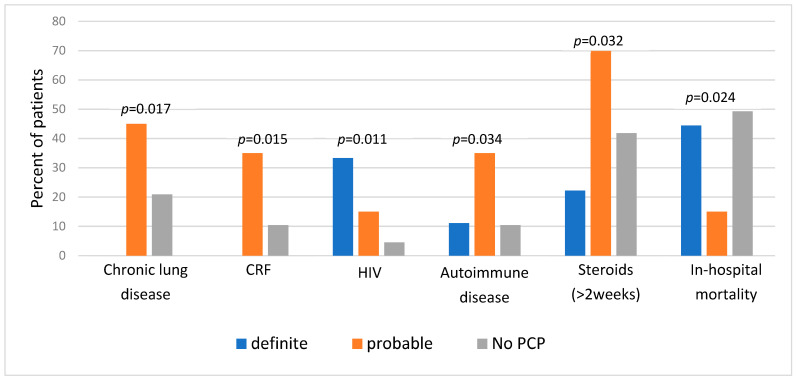
Background comorbidities and outcome of patients with definite, probable, and no PCP (*n* = 96). PCP, Pneumocystis pneumonia; CRF, chronic renal failure; HIV, human immunodeficiency virus.

First, we compared clinical characteristics between the three categories of diagnosis (Figure 1). We found a significantly higher prevalence of specific background comorbidities among patients with “probable” PCP, including chronic lung disease, chronic renal failure, and autoimmune diseases. A history of prolonged steroid therapy (≥two weeks) was also significantly more prevalent in this group. Unsurprisingly, HIV diagnosis was most prominent among patients with “definite” PCP and least present in the “No PCP group”. Regarding outcomes, in-hospital mortality was the lowest in the “probable” group (15% vs. 44.4% and 49.3% in “probable”, “definite”, and “no PCP” groups, respectively, *p* = 0.024).

Next, we compared the “no PCP” group with higher probability PCP cases (29 patients of both “definite” and “probable” groups). In this comparison, only HIV was more prevalent among patients diagnosed with PCP (20.7% in the high probability group versus 4.5% in the “no PCP” group, *p* = 0.02). The in-hospital mortality rates were found to be higher in the “No PCP” group (49.3% in the “No PCP” group vs. 24.1% in the high probability group, *p* = 0.022).

Only 12.5% of the patients in the study group received preventive treatment for PCP (22.2% in the “definite” group, 0% in the “probable” group and 14.9% in the “no PCP” group, *p* = 0.075).

Twenty-nine patients in the study group had positive PCR results, including eight “definite” cases (having positive microscopy), nineteen “probable” cases, and one “No PCP” case. The single remaining “definite” PCP case could not be tested by PCR due to technical issues. Of 85 patients in the control group, only 1 had a positive PCR result for PCP (Table 2).

We evaluated the accuracy of the PCR test in diagnosing PCP. First, we considered the clinical definition of PCP as the gold standard. When examining the study group alone and classifying both probable and definite PCP categories as “true positives”, PCR had a sensitivity, specificity, PPV, and NPV of 93.1%, 98.7%, 93.1%, and 98.7%, respectively. The inclusion of the control group in this analysis increased the PPV without substantially changing the other measures (sensitivity 93.1%, specificity 98.5%, PPV 96.4%, and NPV 97.1%). In addition, we found that the negative likelihood ratio for being PCR positive was 0.71, while the positive likelihood ratio was 46.5. The odds ratio for being PCR positive with a diagnosis of definite or probable PCP was 1012.5 (CI-95%; 136.7–7498.9).

When considering microscopic examination as the gold standard (including only the study group in the analysis), the PCR test had a sensitivity of 89%, specificity of 77%, PPV of 28.6%, and NPV of 98.5%.

Cq values were calculated for 26 of the 29 positive cases. Values ranged from 15.55 to 30, with a mean value of 23.53 ± 4.35, and a median of 22.59. The “definite” group had significantly lower Cq values than the “probable group” (19.83 ± 3.38 vs. 24.96 ± 4, *p* = 0.006, respectively).

**Figure 2:**
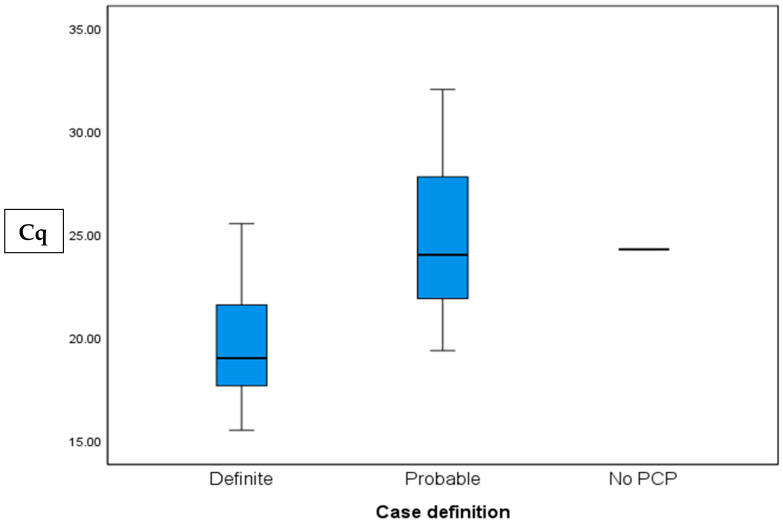
PCR quantification cycle (Cq) values in patients with definite, probable, and no PCP; *p* = 0.006 for definite vs probable.

Only one patient in the “No PCP” group had a positive PCR result, in which case the Cq was 24.32. In the control group; one patient had a positive PCR with a high Cq value of 34.48 (Table 2). A comparison of the Cq values between the diagnosis groups is shown in Figure 2.

**Figure 3:**
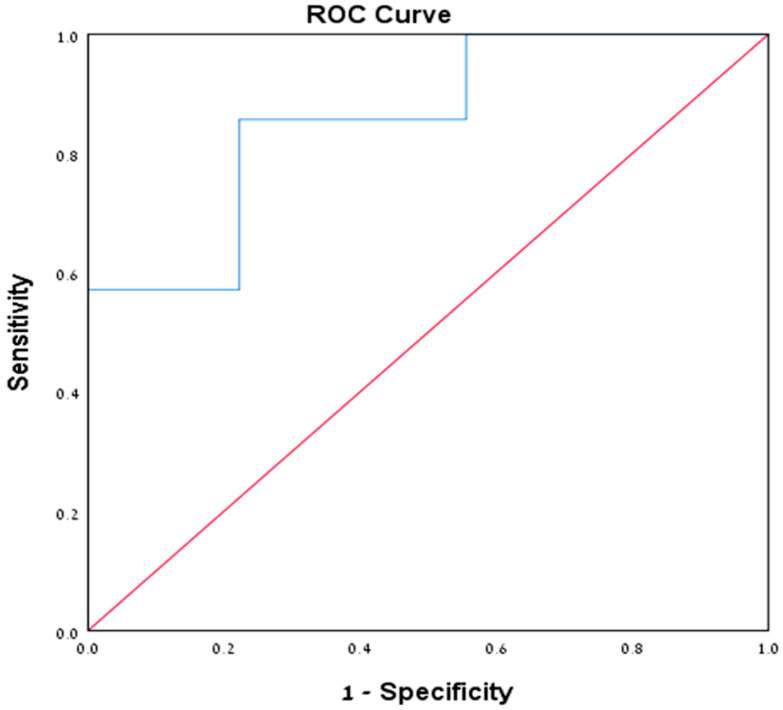
A ROC curve (blue line) of Cq values for the “definite” and “probable” patient groups.

The ROC (receiver operating characteristic) curve (Figure 3), with an AUC (area under the curve) of 0.857, demonstrated that a Cq value of 21.89 was an optimal threshold to discriminate between patients in the “definite” and “probable” groups. Using this value as a cutoff point, the test had a sensitivity of 85.7%, a specificity of 77.8%, PPV of 60%, and NPV of 93.3%. There was no correlation between Cq values and mortality or admission to ICU.

Since there were only two patients with a combination of a positive PCR test and a negative PCP diagnosis, we were not able to draw conclusions regarding the difference in Cq values between disease and colonization with PJ. However, both values were higher than our cutoff value of 21.89.

Alternative diagnoses in the study group: all patients in the study group were evaluated for alternative diagnoses as part of the clinical work up of hospitalized patients. In the ‘No PCP’ group, 48/67 (72%) had alternative diagnoses, most of which were infectious (23 bacterial infections, 11 viral infections, 6 of which were COVID, 3 fungal infections, and 2 mycobacterial infections). Alveolar bleeding and drug-related toxicity were diagnosed in three patients each. In the ‘probable’ group—2/20 (10%) patients had a possible alternative diagnosis—CMV pneumonitis. However, both patients improved clinically despite not receiving treatment for CMV. In the ‘definite’ group, 3/9 (33%) patients had additional diagnoses (CMV, Tuberculosis, and COVID). All three were also diagnosed with AIDS.

## 4. Discussion

Our study evaluated the diagnostic performance of PCR for *Pneumocystis jirovecii* pneumonia (PCP). In this cohort, the PCR assay exhibited high sensitivity (93.1%), specificity (98.7%), PPV (93.1%), and NPV (98.7%). Notably, PCR identified 20 patients with clinically diagnosed PCP who had negative microscopy results, highlighting its possible improved sensitivity compared to the current gold standard.

While the inclusion of the control group increased the PPV to 96.4%, this higher PPV may be less representative of the target population, as the control group was less immunocompromised than the study group. Therefore, the PPV of 93.1% derived from the study group alone may be a more clinically relevant estimate.

Our study has several limitations, the most important being the potential information bias. Although PCR results did not directly inform the clinician’s initial diagnosis (our gold standard), the subsequent categorization of cases, especially regarding treatment decisions, may have been influenced by prior knowledge of PCR results. This raises the possibility of misclassification. For example, patients with false-negative PCR results who did not receive appropriate treatment could have been mistakenly classified as “No-PCP.” While the literature suggests low rates of false-negative PCR results [8], our study design cannot definitively rule out this possibility. However, a recent meta-analysis examining the diagnostic performance of PCR in immunocompromised patients found that PCR negativity can be used to confidently exclude PCP [12]. In addition, our “probable” PCP categorization is consistent with the EORTC/MSG criteria [10], further validating this categorization and providing additional support for the observed sensitivity and specificity.

Another limitation of any study evaluating molecular methods is the limited standardization of the PCR test. There are several different gene targets available for identifying PJ, and currently there is limited standardization among laboratories regarding the specific test method and gene target. However, we used a well-known method [8] which has been evaluated in diverse populations in different regions of the world [11], and has been used in our laboratory consistently for many years.

Our study found an extremely low number of supposedly colonized patients (only two false-positive PCR results), compared with prior studies published in the literature [4,13]. In addition to the lack of standardization of laboratory methods mentioned above, there are other factors stemming from methodological differences that may explain this disparity. There is great variability in colonization rates among different studies and different patient populations [11]. First, it seems that geographical variations may play a role. As shown in people living with HIV, a significantly lower colonization rate was found in Uganda (6%) [14] as opposed to that found in San Francisco (69%) [15]. In addition, immune status and background diseases affect the colonization rate. To elaborate, healthy volunteers were found to have 0% colonization rate [16], compared with 44% in patients treated with steroids [17]. Moreover, patients living with HIV demonstrated an inverse relationship between CD4 counts and colonization rates [18]. Our control group was in fact composed of patients with different background diseases, but there was a relatively low proportion of severely immune-suppressed patients, possibly affecting our results. Importantly, this low colonization rate may support the clinical practice of treating “probable” PCP (positive PCR, negative microscopy).

Due to the low rate of colonization in our study, we were unable to establish a Cq cutoff to distinguish *P. jirovecii* colonization from pneumonia. Nevertheless, the Cq values proved to be accurate in evaluating the fungal burden. The mean Cq value for all patients diagnosed with PCP was 23.53 ± 4.3, lower than the 28 reported in the literature [4]. Within the study group, patients with “probable” PCP had significantly higher Cq values than those with “definite” PCP, consistent with the understanding that low fungal burden leads to both false-negative microscopy and higher Cq values [4]. A Cq value of 21.89 demonstrated potential as a cutoff to differentiate between positive and negative microscopy (sensitivity 85.7%, specificity 77.8%, PPV 60%, NPV 93.3%). Thus, in microscopy-negative patients, a low Cq value, particularly below 21.89, is suggestive of PCP and may indicate a higher fungal burden. However, given the numerous pre-analytical and analytical factors influencing qPCR performance in respiratory samples, these quantitative findings may not be readily transferable and generalizable. Applying our findings in other settings thus requires further validation and verification.

Patients with “probable” PCP had significantly more traditional comorbidities and were more likely to be receiving T-cell suppressive therapy compared to the other groups. Conversely, patients with “definite” PCP had a significantly higher rate of HIV diagnosis. These findings align with the current understanding that HIV-infected individuals present with higher fungal burdens, leading to more frequent positive microscopy results, while non-HIV immunosuppressed patients typically have lower fungal burdens, making microscopic detection more challenging [8].

Alternative diagnoses were significantly more prevalent in the non-PCP group. In contrast, only two patients (10%) in the “probable” PCP group had alternative diagnoses, and both were diagnosed with CMV. However, the clinical improvement of these two patients despite the absence of CMV-directed therapy strongly reinforces our assertion that PCP was the underlying cause of their respiratory illness.

Surprisingly, patients with positive PCR but negative microscopy (“probable” PCP) exhibited the lowest in-hospital mortality among all groups. We hypothesize that this survival advantage stems from two factors: the treatability of PCP compared to other respiratory illnesses in the “no PCP” group, and the lower fungal burden in the “probable” group compared with the “definite” group (as inferred from Cq values), potentially making the infection more amenable to treatment. Our findings suggest that PCR might detect PCP at an earlier stage, with lower fungal burden and potentially a less intense inflammatory response, ultimately leading to improved outcomes, but this requires further clinical investigation. Another potential explanation for the lower mortality in the “probable” PCP group is the misclassification of colonization as active pneumonia. As previously noted, reported colonization rates are higher than those observed in our study [4,13], making misclassification a plausible consideration. However, the fact that most patients in the “probable” group lacked alternative diagnoses and responded clinically to PCP treatment argues against this possibility.

Notably, PCP prophylaxis was strikingly low in the study group, particularly in the “probable” PCP group, where no patients had received prophylaxis prior to their PCP diagnosis. This underscores a clear gap in adherence to current guidelines [13,19,20,21,22], and highlights the urgent need to improve physician awareness and increase implementation of PCP prophylaxis for at-risk patients.

In conclusion, PCR appeared to be an accurate diagnostic tool for PCP diagnosis in our patient cohort. Our experience suggests that a positive PCR result with negative microscopy among patients with a compatible illness could enable early treatment for PCP and potentially improve outcomes. While further research with a larger cohort of colonized patients is needed to validate Cq values for distinguishing colonization from active disease, our findings support the use of PCR for early PCP diagnosis and risk stratification.

## Figures and Tables

**Table 1:** Epidemiological and clinical characteristics of participants.

Variables	Study Group *n* = 96 Number (%)	Control Group *n* = 85 Number (%)	*p* Value
**Background disease**			
Chronic lung disease	23 (24)	26 (34.2)	NS
Hematological malignancy	22 (22.9)	10 (13)	NS
Non-malignant hematological dis	22 (22.9)	3 (4)	*p* < 0.01
Solid malignancy	19 (19.8)	18 (24)	NS
Autoimmune disease	15 (15.6)	6 (8)	NS
Chronic renal failure	14 (14.6)	3 (4)	*p* = 0.02
HIV	9 (9.4)	0	*p* = 0.06
Chronic liver disease	8 (8.3)	1 (1.3)	*p* = 0.04
Bone marrow transplant	8 (8.3)	3 (4)	NS
Solid organ transplant	7 (7.3)	0	*p* = 0.02
Congestive heart failure	6 (6.3)	3 (4)	NS
AIDS in the past	5 (5.2)	0	*p* = 0.04
GVHD	3 (3.1)	1 (1.3)	NS
Dialysis	2 (2.1)	2 (3)	NS
**Immunosuppressive therapy**			
Steroids	44 (45.8)	6 (8)	*p* < 0.001
Steroid use over 2 weeks	41 (42.7)	5 (7)	*p* < 0.001
Chemotherapy	20 (20.8)	2 (3)	*p* < 0.001
**PCP prophylaxis**	12 (12.5)	3 (4)	*p* < 0.048
**Clinical presentation**			
Fever	62 (64.6)	2 (3)	*p* < 0.001
Cough	54 (56.3)	33 (43%)	NS
Shortness of breath	69 (71.9)	29 (38%)	*p* < 0.001
**Outcomes**			
Use of ventilation	57 (62)	0	
ICU stay	53 (55.2)	0	
In hospital mortality	40 (41.7)	0	

HIV, human immunodeficiency virus; AIDS, acquired immunodeficiency syndrome; GVHD, graft-versus-host disease; ICU, intensive care unit.

**Table 2:** PCR positivity rate and Cq values by study group.

Study Group	Total	PCP PCR Positive *n* (%)	Cq Value Mean ± SD
**Definite**	8	8 (100%)	19.83 ± 3.38
**Probable**	20	19 (95%)	24.96 ± 4
**No PCP**	67	1 (1.5%)	24.32
**Control group**	85	1 (1.2%)	34.48

## Data Availability

The original contributions presented in this study are included in the article. Further inquiries can be directed to the corresponding author.
